# Monocular Depth Estimation via Self-Supervised Self-Distillation

**DOI:** 10.3390/s24134090

**Published:** 2024-06-24

**Authors:** Haifeng Hu, Yuyang Feng, Dapeng Li, Suofei Zhang, Haitao Zhao

**Affiliations:** 1 College of Telecommunications and Information Engineering, Nanjing University of Posts and Telecommunications, Nanjing 210003, China; 2 College of Internet of Things, Nanjing University of Posts and Telecommunications, Nanjing 210003, China; 3 Engineering Research Center of Health Service System Based on Ubiquitous Wireless Networks, Ministry of Education, Nanjing University of Posts and Telecommunications, Nanjing 210003, China

**Keywords:** monocular depth estimation, self-distillation, self-supervised learning, normal estimate

## Abstract

Self-supervised monocular depth estimation can exhibit excellent performance in static environments due to the multi-view consistency assumption during the training process. However, it is hard to maintain depth consistency in dynamic scenes when considering the occlusion problem caused by moving objects. For this reason, we propose a method of self-supervised self-distillation for monocular depth estimation (SS-MDE) in dynamic scenes, where a deep network with a multi-scale decoder and a lightweight pose network are designed to predict depth in a self-supervised manner via the disparity, motion information, and the association between two adjacent frames in the image sequence. Meanwhile, in order to improve the depth estimation accuracy of static areas, the pseudo-depth images generated by the LeReS network are used to provide the pseudo-supervision information, enhancing the effect of depth refinement in static areas. Furthermore, a forgetting factor is leveraged to alleviate the dependency on the pseudo-supervision. In addition, a teacher model is introduced to generate depth prior information, and a multi-view mask filter module is designed to implement feature extraction and noise filtering. This can enable the student model to better learn the deep structure of dynamic scenes, enhancing the generalization and robustness of the entire model in a self-distillation manner. Finally, on four public data datasets, the performance of the proposed SS-MDE method outperformed several state-of-the-art monocular depth estimation techniques, achieving an accuracy ($\delta_{1}$) of 89% while minimizing the error (AbsRel) by 0.102 in NYU-Depth V2 and achieving an accuracy ($\delta_{1}$) of 87% while minimizing the error (AbsRel) by 0.111 in KITTI.

## 1. Introduction

Monocular depth estimation is a fundamental technology in the field of computer vision, with broad applications in autonomous driving, 3D reconstruction, and AR. Compared with LiDAR or stereo cameras, it reduces the complexity and cost of the system, attracting great attention from both academia and industry [1,2]. In real-world scenes, humans can perceive space and distance through vision, hearing, and touch. However, for cameras, obtaining 3D structures from a single image is a challenge of uncertainty. Therefore, early works relied on supervised learning, requiring a large amount of training data with depth labels. This limits their widespread application in practice due to the fact that obtaining depth labels usually requires expensive sensors and labor-intensive complex manual annotation. In addition, moving objects in the camera’s field of view will cause the position and shape of objects to be blurred over time. Furthermore, distinguishing static backgrounds from dynamic objects, filtering highly dynamic interference, and maintaining depth consistency remain challenging problems to solve.

To address the issue of a lack of training labels, self-supervised monocular depth estimation has gained significant attention. Self-supervised methods avoid dependence on a large number of depth labels and use the internal structure of the image, geometric relationships, and other self-supervised signals to learn the depth of a scene without explicit depth labels. Some representative self-supervised monocular depth estimation algorithms [3,4,5,6,7] mainly rely on the self-generation task of depth images, where the depth information can be obtained based on the inherent structure of the image and information about different parts of the objects in the image. In the indoor scenario, self-supervised monocular depth network can be divided into a depth estimation network and a pose estimation network, which has become a hot topic in recent years [8,9,10]. However, the aforementioned self-supervised methods mostly rely on the multi-view consistency assumption. Due to the occlusion problem caused by moving objects, it is difficult to ensure depth consistency in dynamic scenes. Sun et al. [11] integrated the normal edges of objects into the constraint factors of depth estimation to yield promising results; their method also incorporated the pseudo-labeling method [12,13,14], which ensured enhanced model generalization without the need for ground truth, and this method enabled the model to perform more robustly when encountering unfamiliar scenarios. In conclusion, previously reported self-supervised methods still suffer from some limitations, such as insufficient accuracy, limited model generalization ability, high computational cost, and insufficient semantic understanding for specific scenes.

To overcome these limitations, researchers have further integrated self-distillation techniques into self-supervised monocular depth estimation. The self-distillation method possesses notable advantages, enabling the depth model to better adapt to various environments and tasks under resource-constrained and data-scarce conditions. The iterative self-distillation method proposed in [15] reduces the reprojection loss for individual pixels in depth information, thereby generating more accurate labels for training. Similarly, Liu et al. [16] minimized large interference in depth information by designing a multi-view check to filter out outliers in the self-distillation iteration process. Although the above methods provide helpful information through the use of a teacher model, they also face the inevitable problem of transmitting blurred or even misleading information to the student model. In dynamic environments, multiple disruptive factors not only make the teacher model fail to converge but also have a negative impact on the student model.

**Figure 1:**
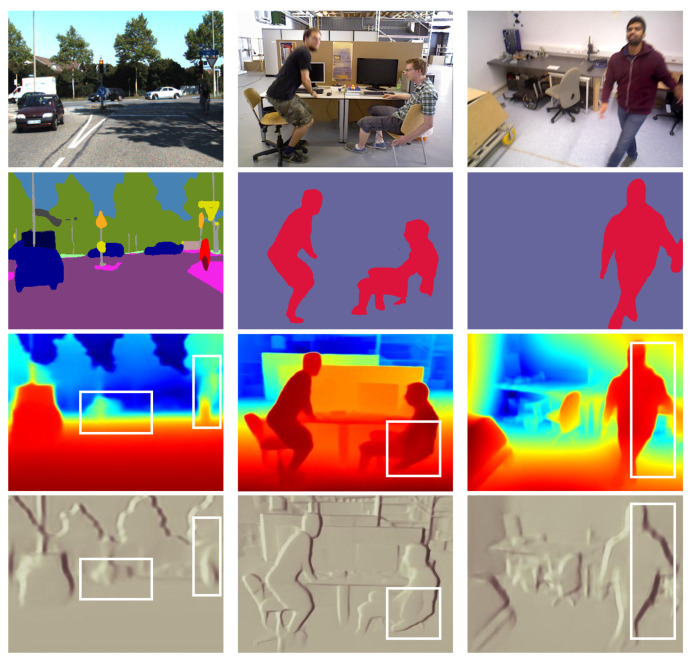
In the KITTI (**left**), BONN (**middle**) and TUM (**right**) datasets, rows 1–4 show raw images, images with region segmentation, images with depth estimation, and images with normal estimation, respectively. White boxes in these images contain moving or overlapped objects, which cause blurred information, degrading the quality of depth estimation.

As shown in Figure 1, there are various problems associated with monocular self-supervised and self-distillation depth estimation in dynamic scenes. First, the movement of objects in the scene results in blurred phenomena in the camera’s view, and overlapped objects can result in inaccurate estimation of their depth order. Secondly, the same object at different depth levels is affected by highly dynamic noise, making edge estimation inaccurate. Therefore, we propose a method of self-supervised self-distillation for monocular depth estimation (SS-MDE) in dynamic scenes. The characteristics of SS-MDE are described as follows: First, during the preprocessing stage, the LeReS [17] network generates pseudo-depth information for each frame. The pseudo-depth matching loss is calculated to gradually reduce dependence by using a forgetting factor. Each frame of the image is downsampled at multiple scales to calculate the smooth loss on multiple scales, enabling the model to better adapt to the field of view of the monocular camera and refine the object edges. Secondly, the sequence of images is fed into the self-supervised network, where the reference frame and the target frame are concatenated for the pose network to estimate a 6-DoF pose. At the same time, a multi-scale encoder-decoder is designed to output the multi-scale disparity of the target frame. With the help of pose estimation, the multi-scale photometric loss is calculated in a self-supervised manner to optimize the depth network. Finally, the current stage of the depth network acts as the student network, and the previous epoch of the depth network acts as the teacher network. The multi-view mask filtering module is used to generate dynamic masks of depth and normal information, filtering out the blurred and uncertain information generated by the teacher network. The student network can better understand the depth structure of dynamic scenes, thereby improving the generalization and robustness of the overall model in dynamic scenes. The contributions of this paper are summarized as follows:

- A self-supervised self-distillation monocular depth estimation (SS-MDE) method is proposed, which uses the existing pseudo-depth network to generate pseudo-depth prior labels and adopts a multi-scale depth network to adapt to the camera’s field of view. At the same time, the multi-scale disparity generated by the multi-scale encoder–decoder is combined with self-supervised information provided by the pose network, ensuring the effective extraction of depth information features. Finally, an iterative self-distillation method with a multi-view mask filtering module is leveraged to improve depth estimation performance in dynamic scenes.
- A forgetting factor is introduced in the calculation of the pseudo-depth matching loss to gradually reduce the dependence on pseudo-depth information, improving the robustness of the depth model. Meanwhile, during the iterative self-distillation process, a multi-view mask filtering module is designed to filter out outliers and inaccurate normal and depth information in the teacher network, enhancing the understanding and generalization capacity of the student network for dynamic scenes.
- The performance of the proposed SS-MDE method is discussed with respect to indoor and outdoor datasets. Multiple comparative experiments were conducted with state-of-the-art methods in dynamic and static scenes, and the results demonstrate the effectiveness and superiority of SS-MDE.

## 2. Related Work

### 2.1. Self-Supervised Monocular Depth Estimation

Due to the difficulty of obtaining depth in practice, self-supervised monocular depth estimation uses methods such as disparity analysis, motion information inference, and geometric constraints to infer the depth information from a single image. Representative methods [5,6,7,18] mainly rely on the precise prediction of optical flow or disparity in cases of texture loss or motion blur. For accurate camera self-motion estimation in complex scenes, Yin et al. [19] enhanced pose estimation by strengthening the surrounding geometric constraints. A new structural loss function was proposed in [20] to optimize single-image depth prediction performance. Recent self-supervised monocular depth estimation methods often divide the model into a depth network model and a pose network model. Godard et al. [21] proposed a minimum projection loss to optimize pose transformation and an automatic masking loss to ignore training pixels that violate camera assumptions. Hoyer et al. [22] combined semantic segmentation with an application scenario of monocular depth estimation and promoted the model’s reasoning performance through knowledge distillation. In dealing with the uncertainty of depth maps, Poggi et al. [23] applied uncertainty to self-supervised monocular depth estimation methods for the first time. For monocular depth estimation problems in dynamic scenes, some researchers use automatic masks to deal with the problem of object occlusion [24,25,26]; by identifying and filtering moving objects in the image, the model is limited to estimating the depth of static areas, thereby improving the accuracy of static object depth estimation. At the same time, the authors of [27] noticed that completely excluding dynamic factors from consideration is also not ideal, so automatic masks were also used in combination with optical flow estimation to enhance the robustness of dynamic object recognition and detection. Adaptive feature fusion [28] also uses local context information capture and image occlusion pre-training technologies to improve the model’s depth estimation performance in texture-sparse areas. Furthermore, Saunders et al. [29] discussed the lighting changes and other issues that monocular depth estimation needs to face under different weather conditions and simulated different weather scenarios for data augmentation to improve the robustness of depth estimation in practical applications. Although existing methods try to consider the complex problems that may be encountered in different scenarios, the above methods either employ image preprocessing for the dataset or overly complicate the model, resulting in poor predictive performance in real environments. Motion blur, occlusion, and noise still exist and are difficult to deal with.

### 2.2. Self-Distillation Monocular Depth Estimation

Self-distillation depth estimation methods usually do not add additional large models as teacher models but choose models similar to the student model for training. Self-distillation can avoid the large training burden incurred by more complex models and has been widely studied in recent years. Pilzer et al. [30] designed a pair of twin depth networks for complementary training of depth information, synchronously transmitting the gain information during the training process. On this basis, in [31], a two-stage network was used to avoid the computational loss incurred by the simultaneous training of the two depth networks, using a selective post-processing method to generate distillation labels. Pan et al. [32] designed a student encoder to extract features from two datasets of indoor and outdoor scenes and introduced dissimilarity loss to separate the feature spaces of different scenes. Weighted multi-task learning [33] was used to learn to minimize the cost of training labels, using self-distillation methods to assist in the training of multi-task learning. Han et al. [34] designed a decoder based on the attention block to enhance the representation of details in the feature map in ensuring global context and used self-distillation’s single-scale photometric loss to improve the performance of the student model. Lv et al. [35] combined the characteristics of transformer and convolutional neural networks to design a depth model and introduced a multi-scale fusion module for the encoder during inference in the self-distillation process to reduce training overhead. In addition, if the three-dimensional or motion structure paradigm used for training of an unsupervised monocular depth model is treated as a probabilistic problem [36], then the uncertainty of the depth prediction of the teacher model can be formulated as a probability distribution for training of the student model. In summary, the above methods focus on efficiently transferring the performance of the teacher model to the student model. However, in a highly dynamic scene (i.e., lighting changes and motion blur), due to the existence of large affected areas in the scenario, the prior estimation of the teacher model may degrade the performance of the student model and even affect the further training of the student model.

## 3. Model and Loss Function

**Figure 2:**
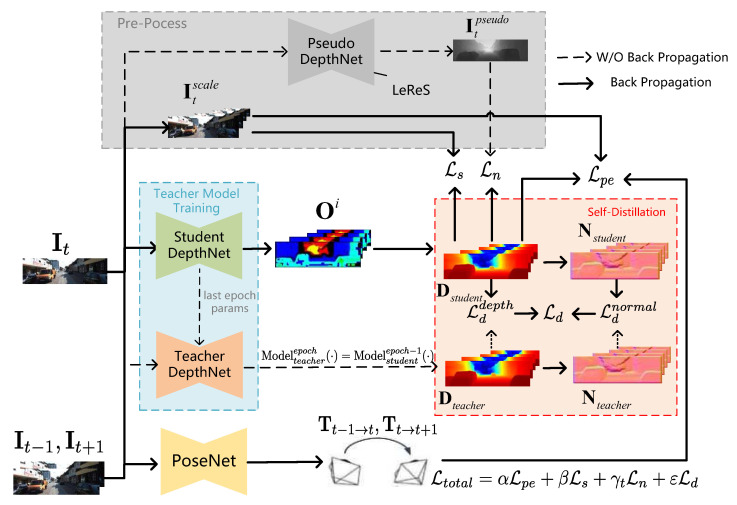
System overview of the proposed SS-MDE.

As shown in Figure 2, the self-supervised self-distillation monocular depth estimation (SS-MDE) method consists of four parts, namely a pseudo-depth network, a teacher depth network, a student depth network, and a pose estimation network. The processing procedure of the SS-MDE method can be described as follows.

In the preprocessing stage, given a consecutive image sequence ($I_{t}$, $I_{t-1}$, $I_{t+1}\in R^{H\times W\times C}$, where $I_{t}$ is the target frame and $I_{t-1}$ and $I_{t+1}$ are both the reference frames), *H*, *W*, and *C* represent height, width and channels, respectively. The target frame ($I_{t}$) is processed as follows:

(1) $$I_{t}^{scale}=\mathrm{Downsample}(I_{t},scale),$$

where $\left\{I_{t}^{scale}\in R^{\frac{H}{2^{scale-1}}\times \frac{W}{2^{scale-1}}\times C}\left|scale=1,2,\ldots ,N\right.\right\}$ is *N* different scale-downsampled images of the current frame, scale is the scaling factor, and bilinear interpolation is used for downsampling. We use LeReS [17] to generate the pseudo-depth ($I_{t}^{pseudo}\in R^{H\times W\times 1}$), which can provide a rough depth estimation of an image without additional training costs. We utilize LeReS [17] to generate pseudo-depth, as shown in [11], which can better comprehend the realistic 3D scene shape and is widely applied in the recovery of 3D scenes. Therefore, LeReS can help to provide relatively accurate depth-supervised information. Then the pseudo-depth ($I_{t}^{pseudo}$) serves as global supervision for the calculation of the pseudo-depth matching loss ($L_{n}$), which is discussed in Section 4.1.

**Figure 3:**
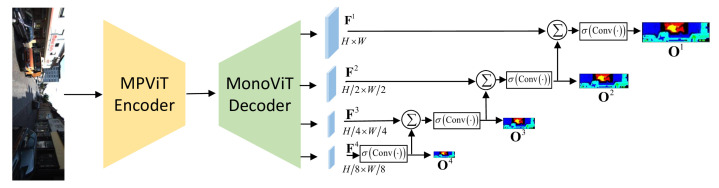
Depth network with encoder and decoder.

In the self-supervised training stage, the encoder of the depth network [37] is designed to combine the local convolutional features and global context-aware property of the transformer for efficient feature extraction. An image is fed into the encoder, and the decoder outputs multi-scale disparities (as shown in Figure 3) for depth prediction. After predicting the depth for multi-scales disparities, the smoothness loss ($L_{s}$) is computed based on the multi-scale target frame ($I_{t}^{scale}$). Meanwhile, the pose estimation network [38] combines the target frame ($I_{t}$) with the reference frames ($I_{t-1}$ and $I_{t+1}$) to predict the corresponding rotation (R) and translation (t); therefore, relative 6-DoF camera pose transformations ($T_{t-1\to t}$ and $T_{t\to t+1}$) can be obtained. Then, the photometric loss ($L_{pe}$) can be computed by warping the output of the pose estimation network with the camera’s intrinsic K. The smoothness loss ($L_{s}$) and the photometric loss ($L_{pe}$) are discussed in Section 4.2.

In the self-distillation stage, the student depth network maintains the same structure as the teacher model. The parameters of the teacher model are derived from the parameters of the student model in the last training epoch, which can be expressed as follow:

(2) $${\mathrm{Model}}_{teacher}^{epoch}\left(\cdot \right)={\mathrm{Model}}_{student}^{epoch-1}\left(\cdot \right).$$

The teacher depth network decoder outputs multi-scale disparity maps to obtain both the depth map (D) and the normal map (N). Subsequently, the well-designed multi-view mask filtering module is introduced; therefore, outliers are filtered from the target view based on both depth and normal maps.

(3) $$M^{depth}=D\left[p\in P_{valid}\right],$$

(4) $$M^{normal}=N\left[p^{\prime }\in P_{valid}^{\prime }\right],$$

where $P_{valid}$ and $P_{valid}^{\prime }$ denote valid sets of pixels without filtration between the target view and the reference view, as discussed in Section 4.3.1 and Section 4.3.2, and the binary masks ($M^{depth}$ and $M^{normal}$) are used to mask regions in depth regions that are heavily influenced by dynamic factors or significant noise, reducing the transfer of misleading depth estimation from the teacher model to the student model. Hence, the student model can focus more on dynamic regions while maintaining accuracy in static regions. Finally, these binary mask modules are employed during the training process of the student model, and the depth self-distillation loss ($L_{d}^{depth}$) and normal self-distillation loss ($L_{d}^{normal}$) are computed. Then, total self-distillation loss ($L_{d}$) can be obtained to iteratively distill knowledge from the teacher model to the student model. A detailed explanation is provided in Section 4.3.

In summary, the overall loss function can be expressed as

(5) $$L_{total}=\alpha L_{pe}+\beta L_{s}+\gamma_{t}L_{n}+\varepsilon L_{d},$$

where α, β, $\gamma_{t}$, and ε are predetermined weights for each loss, adjusting their respective influence during the training process. To prevent the potential accumulation of errors from pseudo-depth supervision during the self-distillation iterations, a dynamically decaying weight ($\gamma_{t}$) with respect to *t* is defined as

(6) $$\gamma_{t}=\gamma_{0}e^{-\mu \left\lfloor \frac{t}{\nu }\right\rfloor },$$

where $\gamma_{0}$ is the initial weight; μ denotes the decay coefficient; ν denotes the interval between training epochs; and ⌊·⌋ represents the floor function, which rounds down to the nearest integer. This helps the model gradually reduce its reliance on pseudo-depth, enhancing the robustness of the overall model. The decay coefficient (μ) is determined by evaluating its impact on performance, as discussed in Section 5.4.1.

## 4. Self-Supervised Self-Distillation Monocular Depth Estimation

### 4.1. Pseudo-Depth Matching Loss

By introducing pseudo-labeling, additional supervision can provide more accurate prediction of single-image depth estimation, enabling a better understanding of the depth structure of the scene and improving the model’s capacity to predict depth information. Specifically, pseudo-labeling can provide additional information to address the label-scarcity issue.

Pseudo-depth [17] is proposed to provide supervised information, which can roughly establish correct depth relationships between objects to improve training efficiency. However, considering its relatively high error and unstable depth predictions in dynamic scenes [39,40], heavy reliance on pseudo-labeling would entail a significant risk, potentially leading to overfitting and poor generalization capacity in practical applications.

In order to overcome this limitation, pseudo-depth is utilized to refine the overall image structure by focusing on object boundary regions [11]. However, this method has a negative impact on the performance of deep models during the self-distillation procedure. This is not only due to the fuzzy boundaries caused by pseudo-depth but also uncertain normal estimates. Calculating an accurate normal map may require high levels of computational resources and easily be affected by noise or uncertainty in highly dynamic environments. Therefore, in order to train the depth network more effectively, the local normal boundaries of pseudo-depth are used to improve depth estimation instead of the global structural normal map.

The Sobel operator [41] is introduced to compute the gradients along the *x* and *y* directions of the image, denoted as Gx=∂z∂z∂x∂x and Gy=∂z∂z∂y∂y, respectively, and the corresponding gradient magnitude ($G=\sqrt{{\left(G_{x}\right)}^{2}+{\left(G_{y}\right)}^{2}}$). Hence, the functional transformation from depth (D) to normal (N) is defined as

(7) N=Ψ(D),

where Nx,y=−Gx,−Gy,1−Gx,−Gy,1Gx2+Gy2+1Gx2+Gy2+1 represents the normal estimation of $\left(x,y\right)$. Based on transformation Ψ(·), the pseudo-depth matching loss [19] can be defined as

(8) $$L_{n}=\frac{1}{N}\sum_{i=1}^{N}\left|n_{i}-n_{i}^{*}\right|,$$

where *N* denotes the total number of pixels in an image, and $n_{i}$ and $n_{i}^{*}$ denote the normal estimation of the *i*-th pixel in $I_{t}$ and $I_{t}^{pseudo}$, respectively.

### 4.2. Multi-Scale Photometric and Smoothness Loss

Depth estimation can achieve better performance with the help of a multi-scale architecture [10,16,37]. We modified the decoder in [10] to adapt to our network architecture, as shown in Figure 3. For the target frame ($I_{t}$) as input, the depth network decoder outputs feature at four scales (${\left\{F^{i}\in R^{\frac{H}{2^{i-1}}\times \frac{W}{2^{i-1}}\times C}\right\}}_{i=1}^{4}$), as shown in [10]. At the minimum scale, $F^{4}$ are processed through convolution and activation operations to obtain a disparity map ($O^{4}\in R^{\frac{H}{8}\times \frac{W}{8}\times 1}$); it is then upsampled and pixel-aligned to match the same scale as $F^{3}$. After merging these two features, convolution and activation operations are applied to output the disparity map ($O^{3}$). Following this description, the decoder outputs disparity maps at four scales (${\left\{O^{i}\in R^{\frac{H}{2^{i-1}}\times \frac{W}{2^{i-1}}\times 1}\right\}}_{i=1}^{4}$) with

(9) $$O^{i}=\mathrm{Sigmoid}\left(\mathrm{Conv}\left(\mathrm{Concat}\left(F^{i},O^{i+1}\right)\right)\right).$$

Equation (7) indicates that it is possible to utilize the multi-scale features to refine low-resolution disparities and compensate them into high-resolution disparities. Here, we apply an activation operation after each merge, which has been proven to further enhance feature acquisition capability. Subsequently, the *i*-th layer multi-scale disparity maps are transformed into the depth map of the corresponding *i*-th layer as follows:

(10) $$D^{i}=\Gamma \left(O^{i}\right),$$

where Dx,y=111dmin−1dmax·Ox,y+1dmax1dmin−1dmax·Ox,y+1dmax. $D\left(x,y\right)$ and $O\left(x,y\right)$ represent the depth value and disparity value, respectively, at $\left(x,y\right)$, and $d_{\min}$ and $d_{\max}$ are predefined depth thresholds. Furthermore, as shown in [7,21], the multi-scale depth maps ($D^{i}$) are combined with the reference frame (${I^{\prime }}_{t}\in \left\{I_{t-1},I_{t+1}\right\}$), the camera intrinsics K, and the pose transformation (T) to obtain ${\left\{{{\tilde{I}}^{i}}_{t}\right\}}_{i=1}^{4}$ as follows:

(11) $${{\tilde{I}}^{i}}_{t}=\mathrm{Warp}\left(\mathrm{Upsample}\left(D^{i}\right),{I^{\prime }}_{t},K,T\right).$$

Then, we define the multi-scale photometric loss ($L_{pe}$) between $I_{t}^{scale}$ and ${{\tilde{I}}^{i}}_{t}$ in Equation (11) as follows:

(12) $$L_{pe}=\frac{\alpha }{4}\sum_{i=1}^{4}\frac{1-\mathrm{SSIM}\left(I_{t}^{scale},{\tilde{I}}^{i}\right)}{2}+\frac{\left(1-\alpha \right)}{4}\sum_{i=1}^{4}{∥I_{t}^{scale}-{{\tilde{I}}^{i}}_{t}∥}_{F},$$

where *i* and scale are scale factors satisfying i=scale and the structural similarity function (SSIM) [42] is used to measure image similarity, we set α to 0.85, as shown in [21].

After that, similar to [7,21], we define the smoothness loss ($L_{s}$) as

(13) $$L_{s}=\frac{1}{4}\sum_{i=1}^{4}\left(\left|\partial_{x}D^{i*}\right|e^{-\left|\partial_{x}I_{t}^{scale}\right|}+\left|\partial_{y}D^{i*}\right|e^{-\left|\partial_{y}I_{t}^{scale}\right|}\right),$$

where $D^{i*}$ represents the normalized depth map, and $\partial_{x}$ and $\partial_{y}$ represent the partial derivatives of the variable in the *x* and *y* directions, respectively.

### 4.3. Multi-View Mask Filtering and Self-Distillation Loss

Although self-distillation can provide supervision during training, the predictions of the teacher model at each pixel are not completely reliable [16]. For instance, the edge regions of target objects often have relatively low confidence, leading to the presence of outliers. This necessitates the adoption of a masking strategy to shield the training process from these potentially harmful outliers. Therefore, we incorporate a multi-view mask filtering module to combine outlier filtering with normal correction, specifically tailored for the decoder structure described above.

#### 4.3.1. Depth Self-Distillation

First, as described in Section 3, we use the parameters of both the teacher and student models to generate the corresponding depth maps (${\left\{D_{teacher}^{i}\in R^{\frac{H}{i-1}\times \frac{W}{i-1}\times 1}\right\}}_{i=1}^{4}$ and ${\left\{D_{student}^{i}\in R^{\frac{H}{i-1}\times \frac{W}{i-1}\times 1}\right\}}_{i=1}^{4}$).

Secondly, we use the teacher model to generate masks for the *i*-th depth map $D^{i}$; we further divide it into a reference depth map ($D_{r}^{i}$) and target depth map ($D_{t}^{i}$) corresponding to reference frames ($I_{t-1}$ and $I_{t+1}$) and the target frame ($I_{t}$). Assume that $p_{t}^{k}=\left(u_{t}^{k},v_{t}^{k}\right)$ is the *k*th pixel of the target view and $p_{r}^{k}=\left(u_{r}^{k},v_{r}^{k}\right)$ is the *k*th pixel of the reference view, satisfying $D_{t}^{i}\left(p_{t}^{k}\right)=z_{t}^{k}$ and $D_{r}^{i}\left(p_{r}^{k}\right)=z_{r}^{k}$. Similar to [16], we transform $p_{t}^{k}$ to the reference view (${\tilde{p}}_{r}^{k}$) in order to obtain ${\tilde{z}}_{r}^{k}$ as follows:

(14) $${\tilde{z}}_{r}^{k}\left[\begin{array}{c} {\tilde{p}}_{r}^{k}\\ 1 \end{array}\right]=KT_{t\to r}K^{-1}z_{t}^{k}\left[\begin{array}{c} p_{t}^{k}\\ 1 \end{array}\right],$$

where $T_{t\to r}$ represents the pose estimation predicted by the pose estimation network, and K denotes camera intrinsic. Similarly, we can obtain ${\tilde{z}}_{t}^{k}$ as follows:

(15) $${\tilde{z}}_{t}^{k}\left[\begin{array}{c} {\tilde{p}}_{t}^{k}\\ 1 \end{array}\right]=KT_{r\to t}K^{-1}z_{r}^{k}\left[\begin{array}{c} p_{r}^{k}\\ 1 \end{array}\right].$$

Based on the above description, we calculate reprojection loss (${\bar{re}}_{t\to r}$ and ${\bar{re}}_{r\to t}$) and geometric loss $({\bar{ge}}_{t\to r}$ and ${\bar{ge}}_{r\to t}$) between the reference view and target view. For the *k*th pixel, losses are defined as follows:

(16) $$\begin{array}{c} {\bar{re}}_{t\to r}=\frac{1}{N}\sum_{k=1}^{N}re_{t\to r}^{k}=\frac{1}{N}\sum_{k=1}^{N}{∥p_{t}^{k}-{\tilde{p}}_{t}^{k}∥}_{2},\\ {\bar{re}}_{r\to t}=\frac{1}{N}\sum_{k=1}^{N}re_{r\to t}^{k}=\frac{1}{N}\sum_{k=1}^{N}{∥p_{r}^{k}-{\tilde{p}}_{r}^{k}∥}_{2},\\ {\bar{ge}}_{t\to r}=\frac{1}{N}\sum_{k=1}^{N}ge_{t\to r}^{k}=\frac{1}{N}\sum_{k=1}^{N}\frac{\left|z_{t}^{k}-{\tilde{z}}_{t}^{k}\right|}{z_{t}^{k}},\\ {\bar{ge}}_{r\to t}=\frac{1}{N}\sum_{k=1}^{N}ge_{r\to t}^{k}=\frac{1}{N}\sum_{k=1}^{N}\frac{\left|z_{r}^{k}-{\tilde{z}}_{r}^{k}\right|}{z_{r}^{k}}, \end{array}$$

where *N* is the total number of pixels in the view.

Finally, the filtering mask ($M_{i}^{depth}\in {\left\{0,1\right\}}^{\frac{H}{i-1}\times \frac{W}{i-1}}$) can be defined as

(17) $$M_{i}^{depth}\left(k\right)=\left\{\begin{array}{c} 0,\;p_{t}^{k}\in P_{valid}^{i},\\ 1,\;\mathrm{else}, \end{array}\right.$$

where $M_{i}^{depth}\left(k\right)$ denotes the *k*th element of the filtering mask for scale *i*, and $P_{valid}^{i}=\left\{p_{t}^{k}\left|re_{t\to r}^{k}<\alpha \min\left({\bar{re}}_{t\to r},{\bar{re}}_{r\to t}\right)\wedge \right.ge_{t\to r}^{k}<\beta \min\left({\bar{ge}}_{t\to r},{\bar{ge}}_{r\to t}\right)\right\}$ represents the effective subset of pixels on single scale *i*. α and β are the hyperparameters to adjust the range of the filtering mask; we set them to 4, as shown in [16].

Then, depth self-distillation ($L_{d}^{depth}$) can be defined as

(18) $$L_{d}^{depth}=\frac{1}{4}\sum_{i=1}^{4}{∥\prod_{M_{i}^{depth}}\left(D_{teacher}^{i}-D_{student}^{i}\right)∥}_{F},$$

where $\prod_{M_{i}^{depth}}$ is the corresponding indicator function formulated as

(19) $${\left[\prod_{M_{i}^{depth}}\left(A\right)\right]}_{k}=\left\{\begin{array}{c} A\left(k\right),\;\mathrm{if}\;M_{i}^{depth}\left(k\right)=1,\\ 0,\;\;\;\;\mathrm{if}\;M_{i}^{depth}\left(k\right)=0. \end{array}\right.$$

Through depth self-distillation loss, the depth estimation generated by the teacher model can be filtered and transferred to the student model, thereby assisting the student model in efficient convergence.

#### 4.3.2. Normal Self-Distillation

As mentioned in Section 4.1, the pseudo-depth cannot guarantee high-precision estimation. In contrast, normal maps can provide more geometric information and the direction of object surfaces, enabling the model to better understand the geometry and curvature. Inspired by [20], normal vectors are nearly parallel to planar regions and always change significantly, making it easier to localize edge positions. Hence, we introduce normal vectors to train the student model effectively.

For the depth maps (${\left\{D_{student}^{i}\right\}}_{i=1}^{4}$ and ${\left\{D_{teacher}^{i}\right\}}_{i=1}^{4}$) of the teacher and student models, we use the transformation (Ψ(·)) in Equation (7) to convert them into normal maps (${\left\{N_{student}^{i}\in R^{\frac{H}{i-1}\times \frac{W}{i-1}\times 3}\right\}}_{i=1}^{4}$ and ${\left\{N_{teacher}^{i}\in R^{\frac{H}{i-1}\times \frac{W}{i-1}\times 3}\right\}}_{i=1}^{4}$). Depth maps typically remain smooth over large areas, with abrupt changes occurring only at specific edge regions. Due to the fact that the edge region is crucial for depth estimation, our aim is to predict the depth of objects’ edge regions rather than texture edges.

To address the issues of sparse supervision in smooth regions and a lack of the depth information of edge regions based on a random sampling method [20], we define a binary mask ($M_{i}^{normal}\in {\left\{0,1\right\}}^{\frac{H}{i-1}\times \frac{W}{i-1}}$) to preserve the edge regions as follows:

(20) $$M_{i}^{normal}\left(k\right)=\left\{\begin{array}{c} 0\;\mathrm{if}\;{∥E_{teacher}^{i}\left(p_{teacher}^{i}\right)-E_{student}^{i}\left(p_{student}^{i}\right)∥}_{2}⩾\tau ,\\ 1\;\;\;\;\;\mathrm{else}, \end{array}\right.$$

where τ is a threshold coefficient, and E(·) can be defined as

(21) $$E\left(p^{k}\right)=\left\{\begin{array}{c} N^{i}\left(p^{k}\right)\;\;p^{k}\in {P^{\prime }}_{valid}^{i},\\ 0\;\;\;\;\mathrm{else}, \end{array}\right.$$

where ${P^{\prime }}_{valid}^{i}=\left\{p^{k}|G^{k}⩾\gamma \max_{1⩽k⩽N}\left(G^{k}\right)\right\}$, as shown in [20], and γ is an adjustable parameter that we set to 0.1. Then, the normal distillation part can be formulated as

(22) $$L_{d}^{normal}=\frac{1}{4}\sum_{i=1}^{4}{∥\prod_{M_{i}^{normal}}\left(N_{teacher}^{i}-N_{student}^{i}\right)∥}_{F},$$

where $\prod_{M_{i}^{normal}}$ is the indicator function defined as

(23) $${\left[\prod_{M_{i}^{normal}}\left(A\right)\right]}_{k}=\left\{\begin{array}{c} A\left(k\right)\;\mathrm{if}\;M_{i}^{normal}\left(k\right)=1,\\ 0\;\;\;\;\mathrm{if}\;M_{i}^{normal}\left(k\right)=0. \end{array}\right.$$

In conclusion, the total self-distillation loss ($L_{d}$) can be described as

(24) $$L_{d}=L_{d}^{depth}+L_{d}^{normal}.$$

### 4.4. Description of SS-MDE

As stated above, the proposed SS-MDE method is designed to construct a unified end-to-end unsupervised self-distillation framework where a self-supervised network generates depth estimation under the supervision of pseudo-depth labels and an iterative self-distillation with filtering mask modules is leveraged to improve the depth estimation performance. As a result, the generalization and robustness of the entire model are boosted. The detailed procedure of SS-MDE is outlined as follows (Algorithm 1):    
**Algorithm 1:** Self-supervised Self-distillation Monocular Depth Estimation (SS-MDE).
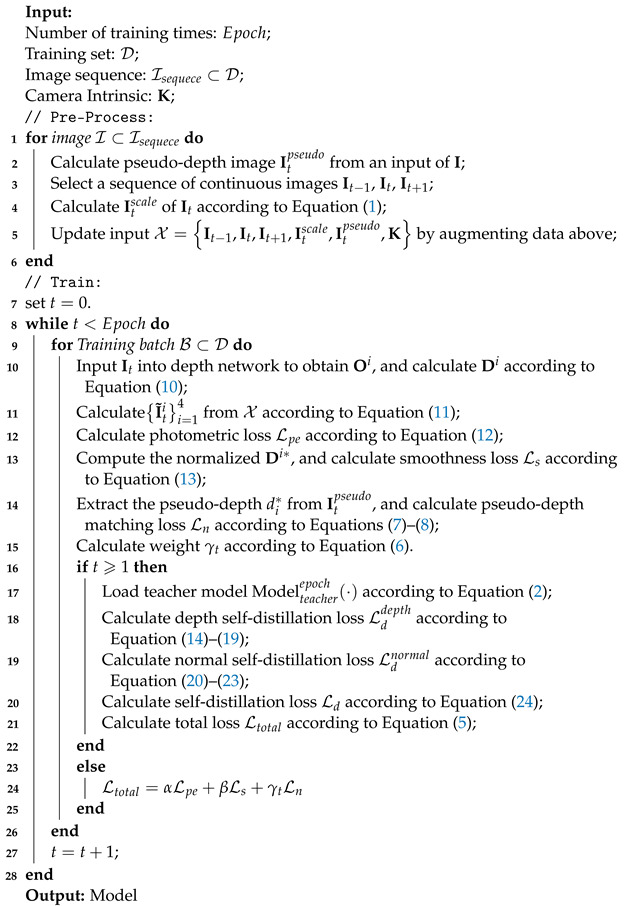


From Algorithm 1, the input sequence (X) is preprocessed before feeding into the self-supervised network. Subsequently, $I_{t}$ is fed into the student depth network to generate depth estimation, and $\left\{I_{t-1},I_{t}\right\}$ and $\left\{I_{t},I_{t+1}\right\}$ are used to calculate pose estimation (T). In the meantime, the parameters of the teacher depth network are frozen, and mask modules ($M^{depth}$ and $M^{normal}$) are introduced to filter out the outliers and uncertain information during the self-distillation process. Finally, the feedforward and back propagation are both employed to optimize the total loss function ($L_{total}$) in a self-distillation manner.

## 5. Experiments and Results

Our SS-MDE method is implemented utilizing the PyTorch framework. All experiments were executed on an NVIDIA GeForce RTX 3090 graphics card with 24,576 MB of memory. Ubuntu 18.04.6 was installed on the served, with Python version 3.8.18, PyTorch version 1.12.1, and CUDA version 11.3.

### 5.1. Experimental Datasets

**KITTI** [43] is one of the most widely used public datasets in the field of autonomous driving. It was created collaboratively by the Karlsruhe Institute of Technology in Germany and the Toyota Technological Institute in the United States. The dataset consists of data collected and synchronized at a frequency of 10Hz using two grayscale cameras, two color cameras, a Velodyne HDL-64E 3D LiDAR, four optical lenses, and a GPS navigation system. In this work, we conducted experiments using image sequences with a resolution of 256×832 [11]. The training set contains 42,440 images, and the test set contains 2266 images.

**NYU-Depth V2** (referred to as **NYUv2**) [44] is a major dataset for depth estimation research in indoor environments. It is provided by New York University and aims to provide rich visual and depth information for depth estimation in indoor scenes. The training set of this dataset contains 26,295 images, and the test set contains 1646 images. However, this dataset predominantly consists of static scenes.

**BONN** [45] is a dataset for depth estimation in dynamic indoor environments. It was constructed by the University of Bonn, Germany, and aims to investigate how to stabilize camera pose estimation in indoor environments with high dynamics. The training set of this dataset contains 23,376 images, and the test set contains 3087 images. The BONN dataset specifically focuses on indoor dynamic scenes, making it suitable for assessing the performance of depth estimation models in such environments.

**TUM** [46] is provided by the Technical University of Munich and comprises a series of datasets used for robot vision and SLAM research. The training set of this dataset contains 9639 images, and the test set contains 1556 images.

### 5.2. Parameter Configuration

During model training, an experiment was conducted for 100 epochs using the AdamW optimizer with an initial learning rate of 0.0001. The hyperparameters of the loss function in Equation (5) were set as follows: α=1, β=0.001, and ε=0.1. In Equation (6), we set the initial weight ($\gamma_{0}$) to 0.1, the decay coefficient (μ) to 0.01, and the interval (ν) to 5. Before training the model, the dataset was divided into multiple batches. The batch size was determined based on the experimental dataset and the memory constraints as follows:

1. For the KITTI dataset, the batch size was set to 4, and each image was resized to a resolution of 256×832;
2. For the NYUv2, BONN, and TUM datasets, the batch size was set to 8, and each image was resized to a resolution of 256×320.

### 5.3. Evaluation Metrics

We used standard depth evaluation metrics, including mean absolute relative error (AbsRel), square relative error (SqRel), root mean squared error (RMSE), logarithmic error ($\log_{10}$), and accuracy under threshold (δ). Given a ground truth ($\hat{d}$), estimated *d*, and the number of pixels in the view (*N*), the above metrics can be defined as follows:

- Absolute relative error (AbsRel) represents the relative error in the average depth estimation for each pixel and is defined as
  (25) $$\mathrm{AbsRel}=\frac{1}{N}\sum_{i=1}^{N}\frac{\left|{\hat{d}}_{i}-d_{i}\right|}{d_{i}}.$$
- Square relative error (SqRel) is similar to AbsRel with the depth difference squared and is defined as
  (26) $$\mathrm{SqRel}=\frac{1}{N}\sum_{i=1}^{N}\frac{{\left({\hat{d}}_{i}-d_{i}\right)}^{2}}{d_{i}}.$$
- Root mean square error (RMSE) represents the specific difference between predicted depth and ground truth. In some cases, it may be influenced by outliers and is defined as
  (27) $$\mathrm{RMSE}=\sqrt{\frac{1}{N}\sum_{i=1}^{N}{\left({\hat{d}}_{i}-d_{i}\right)}^{2}}.$$
- Logarithmic error ($\log_{10}$), similar to the RMSE, compares the logarithm of depth values instead of the actual values. It exhibits good sensitivity to both large and small depth values.
  (28) $$\log_{10}=\frac{1}{N}\sum_{i=1}^{N}\left|\log\left({\hat{d}}_{i}\right)-\log\left(d_{i}\right)\right|.$$
- Accuracy under threshold (δ) examines the performance of a model across different depth ranges rather than focusing solely on the overall average error. It is generally considered that the closer δ is to 1, the better the performance of the model is.
  (29) $$\delta_{j}=\max\left(\frac{{\hat{d}}_{i}}{d_{i}},\frac{d_{i}}{{\hat{d}}_{i}}\right)<1.25^{j}\;j=1,2,3.$$

### 5.4. Results and Discussion

#### 5.4.1. Impact of Decaying Weight $\gamma_{t}$

To prevent the accumulation of errors from pseudo-depth supervision during the self-distillation iteration process, the proposed SS-MDE introduces a dynamically decaying weight ($\gamma_{t}$). To determine the optimal value of the decaying weight in Equation (6), comparative experiments were conducted on NYUv2 and KITTI with various values of $\gamma_{t}$, as shown in Table 1. ↑ means that a higher value is better, ↓ otherwise.

Within the range of decaying weight ($\gamma_{t}$, **0.05–0.3**) in Table 1, the AbsRel and accuracy are almost invariant, indicating that the proposed method is insensitive to the value of $\gamma_{t}$. Additionally, setting $\gamma_{t}$ to a high value may reduce the impact of $L_{n}$, ultimately leading to poor performance. Therefore, we set the decaying weight to **0.1** in the following experiments.

#### 5.4.2. Impact of Weight (ε)

To validate effect of the weight (ε) of the self-distillation loss, we discuss the impact of weight (ε) on the performance of SS-MDE in two datasets. The results of these experiments are presented in Table 2.

From Table 2, we can observe that the model’s overall performance is not sensitive to the value of ε in a large range. However, if the value of ε is set to too low, the performance of SS-MDE deteriorates rather noticeably. In the following experiments, we set ε to 0.1.

#### 5.4.3. Comparison of Sizes of Backbone Networks

In order to verify the impact of different scales of backbone networks on performance, we selected four scales of backbone networks, namely MPViT-Small, MPViT-Xsmall, MPViT-Tiny, and ResNet-18. We compared the performance of the proposed SS-MDE on the KITTI and NYUv2 datasets. The total number of parameters for MPViT-Small is **27.9M**, that for MPViT-Xsmall is **15.4M**, that for MPViT-Tiny is **10.3M**, and that for ResNet-18 is **14.8M** (Note that the overall parameters include those of the designed decoder).

From Table 3, as the number of parameters of the backbone networks increases, **AbsRel**, **SqRel**, $\log_{10}$, and **RMSE** all have a decreasing trend, while $\delta_{1}$, $\delta_{2}$, and $\delta_{3}$ have an increasing trend. The performance of ResNet-18, on the other hand, remains mostly within the range of MPViT-T. It is worth noting that with a similar size, MPViT-X exhibits significantly better performance compared to ResNet-18. Even MPViT-T can achieve comparable performance to ResNet-18 with fewer parameters, indicating that MPViT achieves excellent depth estimation performance in monocular depth estimation tasks. The computational complexity of various models are also presented for further discussion. In the proposed method, the depth network with the MPViT-S backbone has a complexity of **143.7 GFLOPs**, while the depth network with an MPViT-X backbone has a complexity of **74.9 GFLOPs** and the MPViT-T backbone has a complexity of **53.2 GFLOPs**. We can see that the computational complexities of various backbones are consistent with the size of corresponding networks.

#### 5.4.4. Ablation Study

To verify the functionality of the multi-scale decoder (**MSD**), the self-distillation loss ($L_{d}$), the pseudo-depth matching loss ($L_{n}$), and $\gamma_{t}$ in SS-MDE, we carried out ablation experiments on the KITTI and NYUv2 datasets with MPViT-S. Please note that in the following experiment, we used the photometric loss ($L_{p}e$) and the smoothness loss ($L_{s}$) as the baseline, as in [10,16,21]. The results of the ablation experiments are shown in Table 4. “√” means the corresponding module is executed—“×” otherwise.

From Table 4, we can see that the performance of SS-MDE shows a monotonically increasing trend when the corresponding modules are incorporated. This indicates that the multi-scale decoder (**MSD**) can align the output features of MPViT-S, allowing the final depth prediction to better match the local details of the original scale input. Based on the above, the self-distillation loss ($L_{d}$) performs outlier filtering on depth and introduces normal vectors to better distinguish between dynamic and static regions. Moreover, the pseudo-depth matching loss ($L_{n}$) can further refine the edges of objects and local regions. Finally, $\gamma_{t}$ enables the model to enhance adaptability and robustness to dynamic scenes during the self-iterative process, improving the accuracy of depth prediction.

#### 5.4.5. Analyzing Performance

To validate the effectiveness of the proposed SS-MDE method, we compared it with previously reported methods [8,10,11,15,16,19,21,27,30,47,48,49,50,51] on the KITTI, NYUv2, BONN, and TUM datasets. Additionally, to demonstrate the accuracy of identifying dynamic and static regions, we used the semantic segmentation masks proposed in [52] to compare the performance of each method in dynamic and static scenes. In the KITTI dataset, all vehicles and pedestrians are viewed as dynamic objects, while other regions are static objects. In indoor datasets such as BONN and TUM, humans are labeled as dynamic regions. It is noteworthy that all experimental results were obtained based on actual measurements.

Compared with previously reported self-supervised methods, our proposed SS-MDE method outperforms most of the them on the KITTI dataset, as illustrated in Table 5. Furthermore, in Table 6, it can be seen that existing self-supervised methods still cannot surpass the supervised methods. The absence of ground truth limits the performance of the self-supervised methods due to toxic factors such as data noise, occlusions, and camera motion. However, it is noteworthy that recent works such as GasMono [15] and the proposed method have achieved performance comparable with that of VNL [19]. This indicates that self-supervised methods have made significant improvements in monocular depth estimation and are comparable to supervised methods.

On the indoor dataset of TUM with significant dynamic interference, except for $\delta_{1}$, which is slightly worse than SC-DepthV3 [11] in Table 7, the proposed method shows performance improvements compared to other self-supervised methods. In particular, SS-MDE yields significant improvements in RMSE, $\delta_{2}$, and $\delta_{3}$. On the indoor dataset of BONN (Table 8), except for RMSE, which is slightly worse than SC-DepthV3 [11], SS-MDE shows significant improvements in other performance metrics. An overall comparison on both databases indicates that our proposed SS-MDE method performs the best.

As a representative outdoor dataset, KITTI contains multiple vehicles. We considered vehicles in consecutive video sequences moving entities and used them as references for semantic segmentation. In indoor datasets like TUM and BONN, humans become the primary dynamic factor. For comparisons between dynamic and static regions, we selected AbsRel and $\delta_{1}$ as the performance metrics. As shown in Table 9, Table 10 and Table 11, SS-MDE performs slightly worse than the method proposed in [11] but outperforms other methods in the depth estimation of static regions on the TUM and BONN datasets. For the more challenging task of depth estimation of dynamic regions, our proposed method achieves the best performance indicators on each dataset. This indicates that SS-MDE maintains good depth estimation performance in static regions while achieving significant performance gain in depth estimation in dynamic regions.

**Figure 4:**
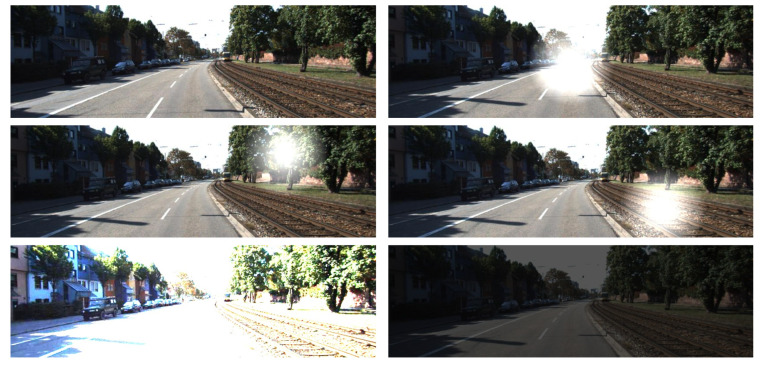
Data augmentation on KITTI.

To validate the performance of the proposed method under various lighting levels, we selected some augmented samples from the KITTI database, as shown in Figure 4.

We generated 40% augmented samples with different lighting levels added to the training set (e.g., local strong light interference, high light intensity, and low light intensity); similarly, we also added augmented samples to the test set. The experimental results are presented in Table 12.

From Table 12, we can observe that the performance of SS-MDE degrades to different degrees in all cases, especially in scenes with high light intensity. However, SS-MDE still exhibits a relatively strong ability to adapt to the environment under different lighting levels despite slight degradation.

#### 5.4.6. Results of Inference

**Figure 5:**
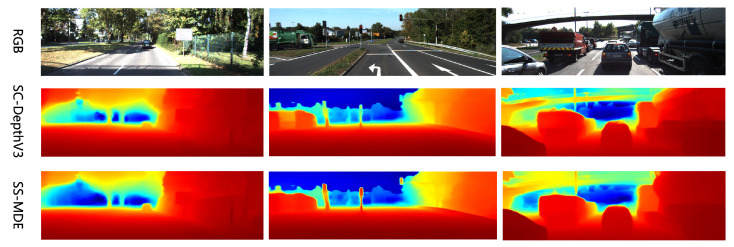
Inference results on KITTI.

**Figure 6:**
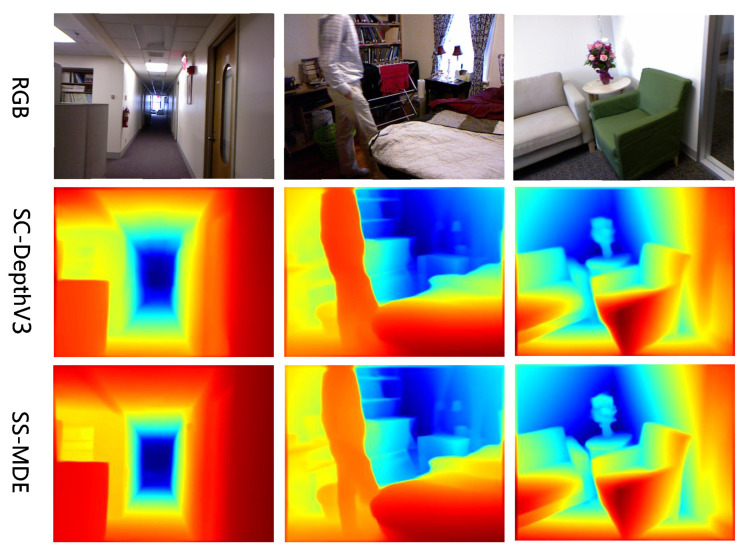
Inference results on NYUv2.

**Figure 7:**
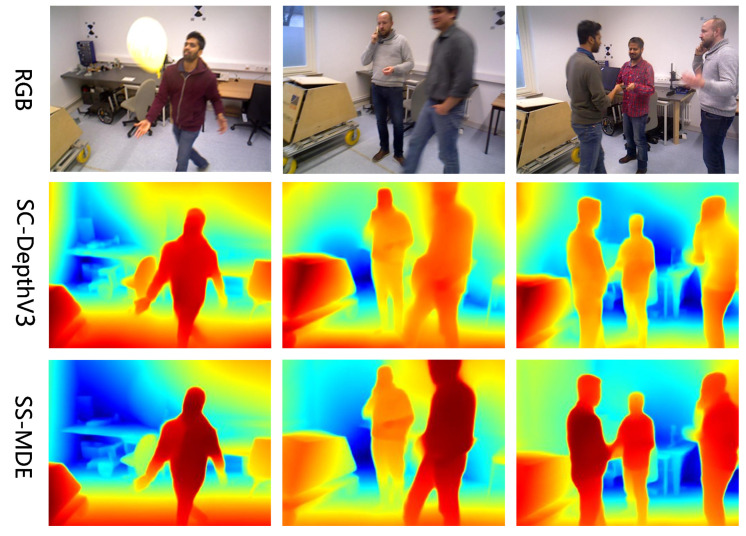
Inferenceresults on BONN.

**Figure 8:**
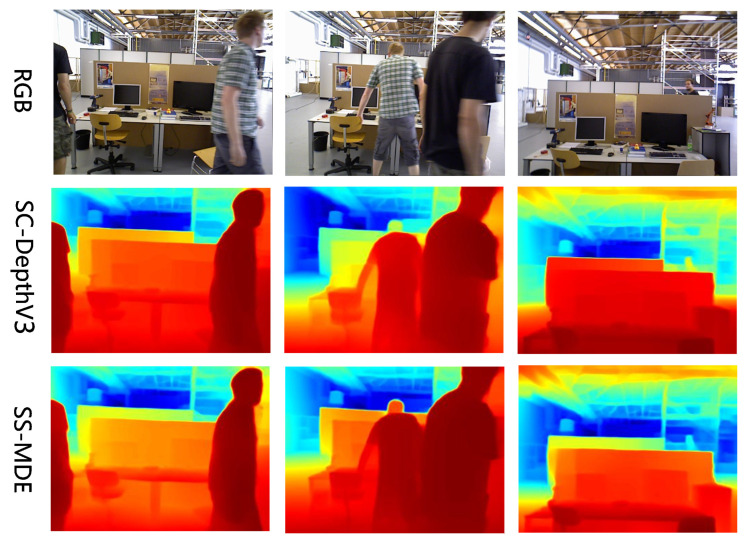
Inference results on TUM.

To validate the inference performance of our proposed method, we conducted inference tests on different scenes from each dataset and compared details in different regions of the views. The results of inference are shown in Figure 5, Figure 6, Figure 7 and Figure 8. In the color mapping, redder regions indicate closer distances to the camera, while bluer regions indicate farther distances from the camera.

Figure 5, Figure 6, Figure 7 and Figure 8 show the depth inference performance of the proposed SS-MDE method. We selected SC-DepthV3 [11] for comparison of depth estimation performance. For the complex depth hierarchy, our method SS-MDE better matches the depth hierarchy of the real-world scenes. By comparing local regions in the depth maps, it can be observed that even in the distant areas, SS-MDE still captures detailed texture structures. Moreover, in depth inference for the highly dynamic regions (such as humans, balloons, cars, etc.), SS-MDE can also achieve excellent results.

## 6. Discussion

As shown in Table 5, Table 6, Table 7, Table 8, Table 9, Table 10 and Table 11, our proposed method achieved superior performance compared to many existing methods. These experimental results show that SS-MDE can capture depth information with higher accuracy in various application scenarios. In summary, the improvement in SS-MDE mainly focus on the following two aspects: a multi-scale encoder–decoder with self-supervised information and an iterative self-distillation with a multi-view mask filtering module. In particular, during the iterative self-distillation process, a multi-view mask filtering module can filter out the outliers and inaccurate normal and depth information of the teacher depth network, improving the feature extraction and generalization capacity of the student network for various dynamic scenes.

At the same time, there still exist some limitations of SS-MDE that need to be addressed and alleviated. For example, the cost of the self-distillation process cannot be neglected, and multi-view mask filtering cannot fully meet the requirements of real applications, especially in some scenarios involving lighting changes and fast-moving objects.

## 7. Conclusions and Future Directions

We propose a self-supervised distillation-based method (SS-MDE) to provide depth estimation in challenging dynamic scenes. We leverage multi-scale encoder–decoder outputs to obtain multi-scale disparities and utilize pose networks to provide effective self-supervised information. Additionally, we employ self-distillation iterations to refine the depth model and incorporate a multi-view mask filtering module to enhance depth understanding and estimation in dynamic scenes. Furthermore, a forgetting factor is introduced to gradually reduce reliance on pseudo-depth, thus enhancing the robustness of the overall model. Finally, comprehensive experiments on four challenging datasets demonstrate the superiority of SS-MDE in depth estimation for dynamic environments. Meanwhile, there still exist some aspects of SS-MDE that need to be improved, e.g., costs incurred by the self-distillation operation should be reduced further. Therefore, we plan to focus on developing more lightweight models that are easier to deploy on resource-constrained platforms.

## Figures and Tables

**Table 1:** Impact of decaying weight ($\gamma_{t}$).

Dataset	$\gamma_{t}$	Error ↓ AbsRel	Accuracy ↑ $\delta_{1}$
NYUv2	0.05	0.112	**0.873**
	0.10	**0.111**	**0.873**
	0.15	**0.111**	**0.873**
	0.20	**0.111**	0.872
	0.25	0.112	0.872
	0.30	0.112	0.871
KITTI	0.05	0.103	0.891
	0.10	**0.102**	**0.892**
	0.15	**0.102**	**0.892**
	0.20	**0.102**	0.891
	0.25	0.103	0.891
	0.30	0.103	0.891

**Table 2:** Impact of weight (ε).

Dataset	ε	Error ↓ AbsRel	Accuracy ↑ $\delta_{1}$
NYUv2	0.05	0.113	0.869
	0.10	**0.111**	**0.873**
	0.15	**0.111**	0.872
	0.20	**0.111**	0.872
	0.25	0.112	0.871
	0.30	0.112	0.871
KITTI	0.05	0.105	0.887
	0.10	**0.102**	0.891
	0.15	**0.102**	**0.892**
	0.20	0.103	0.891
	0.25	0.103	0.891
	0.30	0.103	0.891

**Table 3:** Experimental results of different model sizes. RN-18 denotes ResNet-18, MV-T denotes MPViT-Small, MV-X denotes MPViT-X, and MPViT denotes MPViT-S.

Dataset	Backbone	Error ↓				Accuracy ↑		
		AbsRel	SqRel	$\log_{10}$	**RMSE**	$\delta_{1}$	$\delta_{2}$	$\delta_{3}$
NYUv2	RN-18	0.106	0.682	0.047	4.324	0.885	0.966	0.985
	MV-T	0.107	0.686	0.047	4.438	0.884	0.966	0.986
	MV-X	0.104	0.671	0.046	4.315	0.888	0.968	0.987
	MV-S	0.102	**0.664**	**0.045**	**4.279**	**0.892**	**0.969**	**0.988**
KITTI	RN-18	0.115	0.079	0.050	0.446	0.865	0.970	0.991
	MV-T	0.116	0.080	0.050	0.448	0.864	0.969	0.991
	MV-X	0.114	0.077	**0.049**	0.443	0.867	0.971	0.992
	MV-S	**0.111**	**0.074**	**0.049**	**0.430**	**0.873**	**0.973**	**0.993**

**Table 4:** Ablation experiments on the NYUv2 and KITTI datasets.

Dataset	MSD	$L_{d}$	$L_{n}$	$\gamma_{t}$	Error ↓ AbsRel	Accuracy ↑ $\delta_{1}$
NYUv2	×	×	×	×	0.123	0.859
	√	×	×	×	0.119	0.862
	√	√	×	×	0.114	0.870
	√	√	√	×	0.112	0.872
	√	√	√	√	**0.111**	**0.873**
KITTI	×	×	×	×	0.117	0.871
	√	×	×	×	0.113	0.883
	√	√	×	×	0.107	0.889
	√	√	√	×	0.103	**0.892**
	√	√	√	√	**0.102**	**0.892**

**Table 5:** Comparison experiments of self-supervised methods on the KITTI dataset.

Method	Backbone	Error ↓				Accuracy ↑		
		**AbsRel**	**SqRel**	$\log_{10}$	**RMSE**	$\delta_{1}$	$\delta_{2}$	$\delta_{3}$
PackNet [47]	PackNet	0.109	0.839	0.053	4.696	0.884	0.961	0.981
Monodepth2 [21]	RN-18	0.114	0.848	0.059	4.986	0.869	0.956	0.980
SC-Depth [48]	RN-18	0.118	0.870	0.061	4.997	0.860	0.956	0.981
SGD-Depth [8]	RN-18	0.111	0.857	0.051	4.739	0.884	0.962	0.982
SC-DepthV3 [11]	RN-18	0.118	0.756	0.048	4.709	0.864	0.960	0.984
Refine&Distill * [30]	U-Net	0.114	0.741	0.047	4.696	0.884	0.961	0.984
MonoViT [10]	MV-S	0.114	0.732	0.047	4.654	0.885	0.963	0.984
SRD * [16]	MV-S	0.108	0.725	0.047	4.354	0.887	0.964	0.984
GasMono * [15]	MV-S	0.105	0.713	**0.045**	4.336	0.889	**0.969**	0.985
SS-MDE	MV-S	**0.102**	**0.664**	**0.045**	**4.279**	**0.892**	**0.969**	**0.988**

* Self-distillation method.

**Table 6:** Comparison experiments of supervised methods (first row) and self-supervised methods (second row) on NYUv2.

Method	Error ↓		Accuracy ↑		
	**AbsRel**	**RMSE**	$\delta_{1}$	$\delta_{2}$	$\delta_{3}$
Make3D [3]	0.349	1.214	0.447	0.745	0.897
Stream2Net [53]	0.143	0.635	0.788	0.958	0.991
DORN [50]	0.115	0.509	0.828	0.965	0.992
VNL [19]	**0.108**	**0.416**	**0.875**	**0.976**	**0.994**
MovingIndoor [18]	0.208	0.712	0.674	0.900	0.968
Monodepth2 [21]	0.169	0.614	0.745	0.946	0.987
SC-Depth [48]	0.159	0.608	0.772	0.939	0.982
P2Net [49]	0.150	0.561	0.796	0.948	0.986
SC-DepthV2 [48]	0.138	0.532	0.820	0.956	0.989
MonoIndoor [51]	0.134	0.526	0.823	0.958	0.989
SC-DepthV3 [48]	0.123	0.486	0.848	0.963	0.991
GasMono * [15]	0.113	0.459	**0.874**	**0.973**	0.992
SS-MDE	**0.111**	**0.430**	0.873	**0.973**	**0.993**

* Self-distillation method.

**Table 7:** Comparison experiments of self-supervised methods on BONN.

Method	Backbone	Error ↓				Accuracy ↑		
		**AbsRel**	**SqRel**	$\log_{10}$	**RMSE**	$\delta_{1}$	$\delta_{2}$	$\delta_{3}$
Monodepth2 [21]	RN-18	0.565	0.103	0.059	2.337	0.352	0.591	0.728
SC-Depth [48]	RN-18	0.272	0.096	0.055	0.733	0.623	0.858	0.948
SC-DepthV2 [54]	RN-18	0.211	0.095	0.054	0.619	0.714	0.873	0.936
SC-DepthV3 [11]	RN-18	0.126	**0.085**	**0.051**	**0.379**	0.889	0.961	0.980
SS-MDE	MV-S	**0.125**	**0.083**	**0.051**	0.401	**0.891**	**0.969**	**0.986**

**Table 8:** Comparison experiments of self-supervised methods on TUM.

Method	Backbone	Error ↓				Accuracy ↑		
		**AbsRel**	**SqRel**	$\log_{10}$	**RMSE**	$\delta_{1}$	$\delta_{2}$	$\delta_{3}$
Monodepth2 [21]	RN-18	0.312	0.064	0.088	1.408	0.474	0.793	0.905
SC-Depth [48]	RN-18	0.257	0.056	0.081	0.283	0.616	0.814	0.909
SC-DepthV2 [54]	RN-18	0.223	0.053	0.079	0.282	0.643	0.862	0.932
SC-DepthV3 [11]	RN-18	0.163	0.051	0.075	0.265	**0.797**	0.882	0.937
SS-MDE	MV-S	**0.159**	**0.049**	**0.073**	**0.217**	0.794	**0.923**	**0.974**

**Table 9:** Comparison experiments in dynamic and static regions on KITTI.

Method	Dynamic		Static	
	**AbsRel**↓	$\delta_{1}↑$	**AbsRel**↓	$\delta_{1}↑$
PackNet [47]	0.208	0.737	0.099	0.901
Monodepth2 [21]	0.187	0.731	0.104	0.884
SC-Depth [48]	0.242	0.698	0.108	0.878
SGD-Depth [8]	0.209	0.728	0.101	0.899
SC-DepthV3 [11]	0.205	0.703	0.108	0.881
MonoViT [10]	0.184	0.743	0.102	0.894
SRD [16]	0.181	0.753	0.098	0.901
GasMono [15]	0.179	0.761	0.097	0.902
SS-MDE	**0.175**	**0.766**	**0.095**	**0.904**

**Table 10:** Comparison experiments in dynamic and static regions on BONN.

Method	Dynamic		Static	
	**AbsRel**↓	$\delta_{1}↑$	**AbsRel**↓	$\delta_{1}↑$
Monodepth2 [21]	0.474	0.172	0.594	0.383
SC-Depth [48]	0.704	0.166	0.180	0.715
SC-DepthV2 [54]	0.488	0.247	0.152	0.803
SC-DepthV3 [11]	0.220	0.720	**0.102**	**0.931**
SS-MDE	**0.190**	**0.760**	0.114	0.915

**Table 11:** Comparison experiments in dynamic and static regions on TUM.

Method	Dynamic		Static	
	**AbsRel**↓	$\delta_{1}↑$	**AbsRel**↓	$\delta_{1}↑$
Monodepth2 [21]	0.431	0.348	0.262	0.526
SC-Depth [48]	0.512	0.274	0.176	0.715
SC-DepthV2 [54]	0.283	0.494	0.206	0.686
SC-DepthV3 [11]	0.165	0.796	**0.171**	**0.780**
SS-MDE	**0.154**	**0.804**	0.173	0.775

**Table 12:** Impact of various lighting levels (lighting level is divided into three categories, namely local strong light, high light intensity, and low light intensity) No data augmentation means normal lighting level, i.e., the baseline.

Method	Dynamic		Static	
	**AbsRel**↓	$\delta_{1}↑$	**AbsRel**↓	$\delta_{1}↑$
Local strong light	0.169	0.771	0.195	0.728
High light intensity	0.204	0.687	0.231	0.634
Low light intensity	0.176	0.745	0.193	0.732
No data augmentaion	**0.154**	**0.804**	**0.173**	**0.775**

## Data Availability

Data available in a publicly accessible repository.

## Abbreviations and Symbols

The following abbreviations are summarized to facilitate searches:

MDE	Monocular Depth Estimation
AR	Augmented Reality
SSIM	Structural Similarity
DoF	Degree of Freedom
${\mathrm{Model}}_{student}^{epoch-1}$	The student model from the previous epoch
$I_{t}^{scale}$	Image after scaling transformation
$T_{r\to t}\in R^{4\times 4}$	The transformation matrix from the reference view to the target view
$T_{t\to r}\in R^{4\times 4}$	The transformation matrix from the target view to the reference view
$E\in R^{H\times W\times C}$	Edge map
